# Application of Bio-Waste Modified by Ionic Liquids in Epoxy Composites—From Preparation to Biodegradation

**DOI:** 10.3390/molecules30183714

**Published:** 2025-09-12

**Authors:** Anna Sienkiewicz, Piotr Czub

**Affiliations:** Department of Chemistry and Technology of Polymers, Cracow University of Technology, Warszawska Str. 24, 31-155 Cracow, Poland; piotr.czub@pk.edu.pl

**Keywords:** ionic liquids, soybean oil, wood filler, epoxy resin, biodegradation, biopolymers

## Abstract

The research presented in this manuscript aimed to conduct complex studies on epoxy composites filled with modified biowaste. This work discusses the entire process: first, the preparation and analysis of oak waste flour used as an additive to epoxy materials based on Epidian 6; then, obtaining and characterizing epoxy composites containing 5 wt.% of biowaste; and finally, the determination of the influence of wood filler and modification performed using selected ionic liquids (tetradecyltrihexylphosphonium bis(trifluoromethylsulfonyl)amide (IL-1), tetradecyltrihexylphosphonium bis(2,4,4-trimethylpentyl)phosphinate (IL-2), and 1-ethyl-3-methyl-imidazolium bis(trifluoromethylsulfonyl)imide) (IL-3)) on the susceptibility of epoxy composite to the enzymatic degradation. The enzymatic degradation was performed for four weeks using the lipase enzymes (Porcine Pancreas and Rhizopus Oryzae). The epoxy composition EP6-WF_IL-2, containing 5 wt.% of wood flour modified with IL-2, was characterized by the best mechanical parameters in terms of bending strength and flexural modulus (65.64 MPa and 1855.3 MPa, respectively). During enzymatic biodegradation, the lowest susceptibility to enzymatic degradation, regardless of the incubation conditions, was observed in samples of EP6-WF_IL-3 epoxy composition containing wood flour modified with methyltrioctylammonium bis(trifluoromethylsulfonyl)imide.

## 1. Introduction

Epoxy resins, one of the most versatile thermosetting polymers, were introduced to the industrial market in the first half of the 20th century [1]. Due to their advantageous and unique properties compared to other synthetic materials, they have found wide application in various branches of industry, including electrical engineering, electronics, automotive, and construction industries [2,3,4].

According to the definition, epoxy resins are compounds (pre-polymers) that contain more than one epoxy group in their molecules. Depending on their molecular weight, they occur as viscous liquids or thermoplastic solids that become insoluble and infusible after crosslinking. Crosslinked materials are commonly called ‘epoxy resins’, even though after the hardening process, they no longer contain oxirane groups. The wide range of applications of epoxy resin-based materials results from their excellent properties after curing (mechanical strength, resistance to weathering, low water absorption, high thermal stability, good dielectric properties, and high adhesion to various materials, including metals, glass, concrete, ceramics, and other polar plastics).

Although cured epoxy resins are characterized by quite good resistance to deformation and elasticity, they are brittle and inflexible materials, with relatively low impact strength and elongation. These parameters significantly determine the occurrence of various internal stresses, leading to cracking of these materials [5,6]. Due to the aforementioned limitations of the polymer matrix, epoxy resins are often subjected to various modifications. One of them is the production of composite materials based on epoxy resins using fillers of both organic and inorganic origin [7,8,9].

In recent years, various natural fibers have been widely used in epoxy composites, including flax, hemp, and bamboo fibers [10,11]. They are gradually replacing and displacing synthetic fillers, such as glass, carbon, and metal fibers. Their versatile use results from their complete biodegradability and thus non-toxic nature for the natural environment, wide availability, reduced production costs, and satisfactory mechanical properties [12]. The properties of natural fillers introduced into polymer compositions are widely described in the literature. Simultaneously, it was found that the mechanical parameters of epoxy composites containing fillers of natural origin depend on the type of fibers, the nature of the polymer matrix, and the bonding surface between the fibers and the matrix [13].

High cellulose content in natural fibers affects their significant hydrophilicity. That is why it is difficult to obtain a stable bond between a hydrophobic polymer and a hydrophilic natural fibre in the wood–polymer composite materials. Additionally, lignocellulosic fillers are more likely to aggregate due to the formation of hydrogen bonds between natural additive molecules. This leads to a limitation in their wide application in the plastics market. Natural fillers exhibit poor dispersion ability within the polymer matrix and relatively high moisture sorption, resulting in a greater tendency for deterioration of mechanical properties. Hence, to improve the compatibility of natural origin fillers with the hydrophobic polymer matrix, additives are often subjected to various chemical, physical, and biological modifications [14,15,16]. Physical treatments mainly include corona or plasma treatments [17,18]. Among methods of chemical modifications, the most mentioned are mercerization [19], silanization [20], acetylation [21,22], and treatment with potassium permanganate [23] or organic peroxides [24]. All of these reactions are generally based on the addition (or elimination) of specific functional groups to the surface of the fiber, resulting in enhanced thermal stability and improved wettability. Among the chemical modifications of lignocellulosic fillers, there are also mentioned applications of ionic liquid, also called ‘solvents of new generation’ or ‘green solvents’ [25]. Ionic liquids are organic salts with bulky organic cations and organic/nonorganic anions. The melting temperature of ionic liquids does not exceed 100 °C [26], and due to their properties, ionic liquids are used in a wide variety of applications [26], including applications in various studies on lignocellulosic materials. To name only a few of them, these include studies on the influence of the structure of ionic liquids on the process of dissolution of cellulose [27], studies on the application of ionic liquids as a reaction medium during the process of cellulose functionalization [28,29], or studies on the utilization of ionic liquids as direct modifiers of lignocellulosic materials [30].

The aim of the studies discussed within the present manuscript was the examination of the effect of the application of chemically modified waste oak flour from parquet flooring processing in wood–epoxy composites based on low-molecular-weight epoxy resin Epidian 6. The conducted research included (1) an analysis of the influence of selected ionic liquids on the modification of the properties of waste wood flour, (2) a comparison of the influence of the chemical modification on the improvement of mechanical properties concerning the composite containing unmodified wood flour, and (3) an investigation into the possible influence of the use of ionic liquid modified wood flour on the increase in susceptibility to enzymatic degradation.

## 2. Results and Discussion

### 2.1. Chemical Modification of Waste Wood Flour

**Figure 1:**
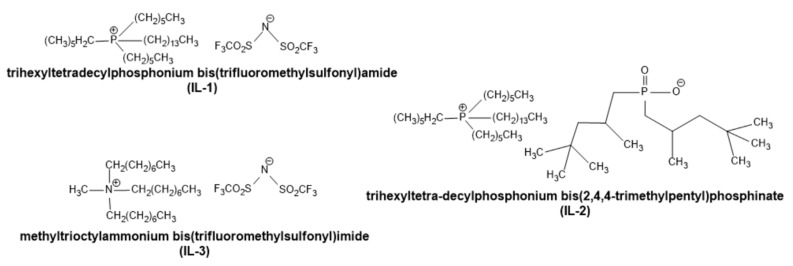
Chemical structure of ionic liquids used for the modification of waste wood flour.

To enhance the compatibility of the natural filler with the hydrophobic epoxy matrix, the waste wood flour was chemically modified using an ionic liquid. The modification of wood waste was carried out using three ionic liquids: trihexyltetradecylphosphonium bis(trifluoromethylsulfonyl)amide (IL-1), trihexyltetradecylphosphonium bis(2,4,4-trimethyl-pentyl)phosphinate (IL-2), and methyltrioctylammonium bis(trifluoromethylsulfonyl)imide (IL-3) (Figure 1). IL-1 is an ionic liquid, available, e.g., under the trade name CYPHOS^®^IL 109, commonly used in electrodeposition of biosensors in electrochemistry and applications in synthetic chemistry, as well as in studies of interactions of ionic liquids with fluorinated alkanes [31]. IL-2 is an ionic liquid that is a polar solvent, which is available, for example, under the trade name CYPHOS^®^IL 104, and is used, among others, as an extractant for removing dibenzothiophene from liquid fuels by extractive desulfurization (EDS) [32]. In contrast, IL-3 belongs to the quaternary ammonium salts, which, due to their properties, are used in phase transfer and metal extraction, including the removal of metals from hydrochloric acid solutions [33].

**Figure 2:**
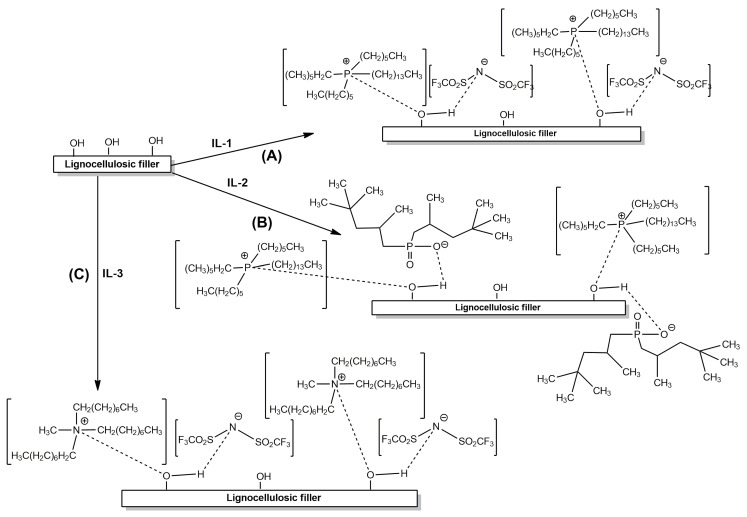
Chemical modification of wood flour with appropriate ionic liquids: IL-1 (**A**), IL-2 (**B**), and IL-3 (**C**).

The ionic liquid was added to the fractionated, dried wood waste in an amount of 0.1 g per 1 g of wood flour. Reagents were thoroughly mixed with a baguette, and then ethanol was added, mixed, and incubated in a covered beaker for another 3 h at the lab temperature. Next, the modified wood waste was filtered on a Büchner funnel under reduced pressure and dried in a dryer at 60 °C for 24 h. Figure 2, prepared using ChemDraw Ultra 12.0 software, presents schematically the probable interaction of the ionic liquids (IL-1, IL-2, and IL-3) with lignocellulosic waste.

As a result of modifying wood flour with IL-1 (Figure 2A), the filler structure became more granular and loose, yet the grain size remained quite uniform. The modified waste WF-IL-2 (Figure 2B) appeared to be significantly less loose and more compact, compared to unmodified wood waste or oak flour obtained in the case of previously performed modification using ionic liquid IL-1. After the modification of wood flour with IL-3 (Figure 2C), significant delamination of the filler was observed. The wood particles became significantly larger and non-uniform, compared to the particles of the oak waste before modification.

**Figure 3:**
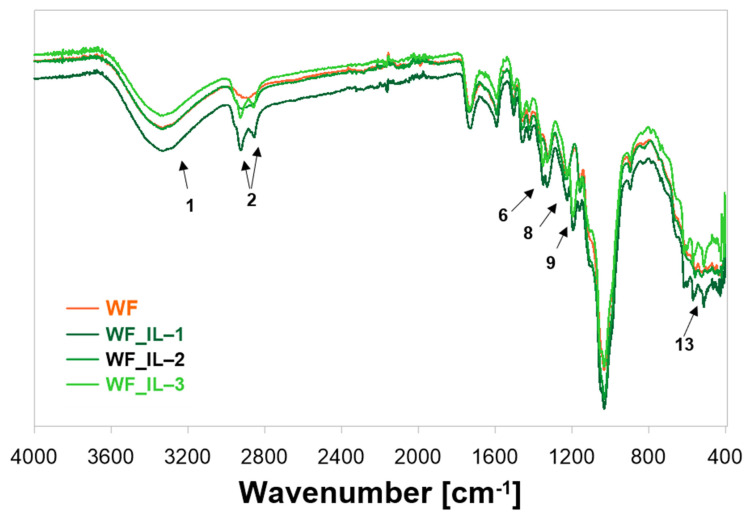
FT-IR spectrum of unmodified and chemically modified waste oak flour using ionic liquid (WF—unmodified waste oak flour, WF_IL-1—waste oak flour modified using trihexyltetradecylphosphonium bis(trifluoromethylsulfonyl)amide, WF_IL-2—trihexyltetradecylphosphonium bis(2,4,4-trimethylpentyl)phosphinate, and WF_IL-3—methyltrioctylammonium bis(trifluoromethylsulfonyl)imide).

In the next stage of the research, the chemical structure of the obtained fillers was analyzed. FT-IR spectra (Figure 3, Table 1) were recorded for unmodified waste wood flour (WF) and wood flour modified with ionic liquids IL-1 to IL-3.

In recorded spectra, presented in Figure 3 and Table 1, characteristic broad bands (1), corresponding to stretching vibrations of the -OH group and hydrogen bonds of hydroxyl groups in the range of about 3100–3600 cm^−1^, are visible. Signal (2) at around 3000–2800 cm^−1^ is most probably the characteristic band of stretching vibrations -C-H from the -CH and -CH_2_ groups of cellulose and hemicellulose [34]. In turn, signal (3) at v = 1724 cm^−1^ can be assigned to the carbonyl group -C=O of stretching vibrations of the carboxyl group in lignin or the ester group in hemicellulose [35]. Signals (4) of relatively low intensity at v = 1592 cm^−1^ and 1470 cm^−1^ are attributed to the vibrations of the -C=C- skeleton of the aromatic ring in the lignin structure. On the other hand, the signal at v = 1425 cm^−1^ (5) is associated with the symmetric bending of the -CH_2_ group present in cellulose [36]. The next two signals, at v = 1314 cm^−1^ and v = 1363 cm^−1^, marked in the spectrum as (6), are characteristic of the bending vibrations of the -C-H and -C-O groups of polysaccharides [37]. In turn, those recorded at v = 1227 cm^−1^ (7) were most likely the stretching vibrations of the -C-O acetyl group in lignin. A signal recorded at 1200 cm^−1^ marked as (8) in the case of wood flour WF_IL-1 is probably related to stretching -C-O-C of the amide group, while (9) in WF-IL-3—bending H-C-C and H-C-N of the imide ring. The next two signals at v = 1106 and v = 1055 cm^−1^ (10) are related to the stretching vibrations of the -C-O-C- pyranose ring, occurring in polysaccharides [38]. Additionally, it is worth mentioning that the signal at v = 1028 cm^−1^ (11) can be assigned to the stretching vibrations of the -C- O hydroxyl and ether groups in cellulose [34]. In turn, the signal of relatively low intensity at v = 891 cm^−1^ (12) is related to the presence of β-glycosidic bonds in monosaccharides, and the one at v = 560 cm^−1^ (14) corresponds to the bending vibrations of the -C-OH group [39]. Within the spectrum of WF_IL-1, in the region of stretching vibrations of the bond of the -O-H group (1), a slightly higher intensity of the band was observed, which may suggest additional overlap with signals characteristic of the -N-H bond, occurring in IL-1, which was used to modify wood flour. Moreover, for WF_IL-1 and WF_IL-3, in the region of stretching vibrations of valence bonds -C-H (2), two signals of higher intensity were noted, which can be associated with stretching vibrations of the carbonyl bond -C=O for the amide or imide group, respectively. In turn, the signal at 1354 cm^−1^ (6) in the spectrum of WF_IL-3—wood flour modified with IL-3, can be attributed to the -C-N stretching vibrations of the imide group. In addition, the signal at 1185 cm^−1^ is characteristic of the bending vibrations of the H-C-C and H-C-N groups of the imide ring (9). On the other hand, the bands in the range of 500–620 cm^−1^ can be attributed to vibrations that correspond to deformations of the imide ring (13) [40]. In the case of the spectrum WF_IL-2, no significant changes were observed compared to the signals recorded for pure wood waste.

**Figure 4:**
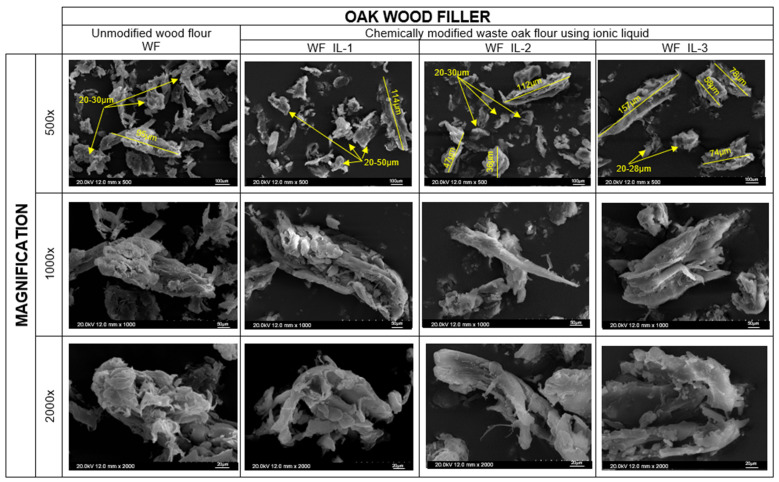
The influence of chemical modification on the morphology of wood flour particles.

The chemical modification using ionic liquids had an impact both on the chemical structure and the morphological appearance of oak flour particles (Figure 4). The surface of unmodified wood flour is ragged and irregular, with numerous long hanging shreds, which are most likely residual post-processing of wood. The measured dimensions of individual wood particles of unmodified oak flour were within the range of 20–30 µm, with a few larger particles in the range of 90 µm. Due to the modification, an enlargement of wood particles was observed. Among all modified wood particles, the largest fragments were observed in the case of modification using IL-3. Most of the particles were about 50–80 µm or larger, up to 160 µm. It is worth recalling that the obtained results of microscopic analysis confirm the previous organoleptic observations. It was mentioned there that organoleptically, WF_IL-1 had the closest form to unmodified wood flour, while WF_IL-2 appeared to be more compact, and WF_IL-3 was characterized by significant delamination of grains, which appear larger and non-uniform, compared to the particles of the oak waste before modification.

### 2.2. Obtaining and Testing the Selected Properties of Epoxy Composites with Wood Waste Flour Modified by Ionic Liquids

In the next stage of the research, epoxy compositions containing unmodified or chemically modified natural waste were prepared. For this purpose, appropriately prepared wood flour in the amount of 5% by weight of the total composition and BYK A530 deaeration agent were added to epoxy resin (EPIDIAN^®^6 with epoxy number EV = 0.537 mol/100 g resin). The whole mixture was mixed using a mechanical mixer for 30 min, and then the amine hardener IDA was added. The mixture, containing epoxy resin with wood filler and a calculated amount of hardener, was again intensively mixed with a baguette until a homogeneous consistency was obtained. Then the mixture was deaerated at a pressure of 0.6 MPa for 3 min, poured into Teflon molds, and left to harden at room temperature for 24 h. After this time, the finished shapes in the form of paddles, beams, and rollers were removed from the molds, seasoned at room temperature, and then crosslinked at 80 °C for the next 24 h. In this way, five epoxy compositions were obtained, including a reference composition without wood filler, compositions containing unmodified wood flour, or chemically modified wood flour (Table 2).

The obtained epoxy compositions were subjected to testing of selected mechanical properties, such as static tensile strength, bending strength, compressive strength, Young’s modulus, Charpy impact strength, and Rockwell hardness.

**Figure 5:**
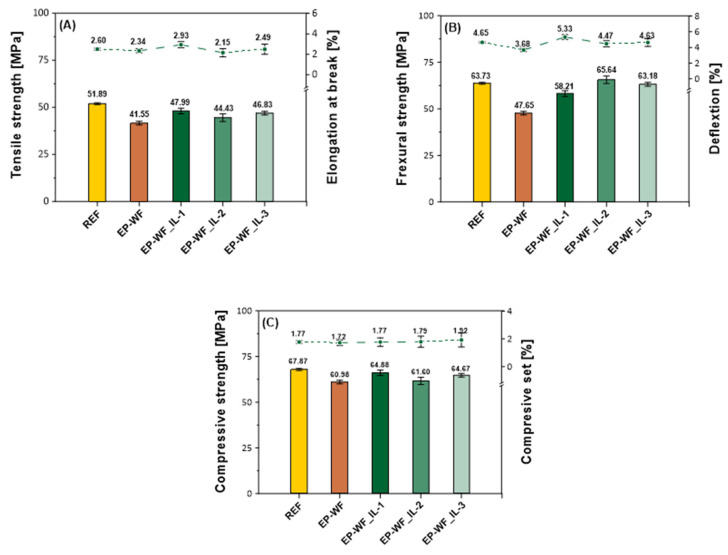
Mechanical properties of epoxy compositions containing unmodified or chemically modified natural waste: tensile strength and elongation at break (**A**), flexural strength and deflection (**B**), compressive strength and compression set (**C**).

The introduction of unmodified wood flour to the composition based on low-molecular-weight epoxy resin resulted in the greatest reduction in the tensile strength value (Figure 5A and Table 3). Compared to the reference composition, characterized by a tensile strength of 51.89 ± 3.99 MPa, a 19.9% decrease in the value was recorded for the EP6-WF composition. The highest Young’s modulus value was also recorded for the reference composition (2441.2 MPa). Among the compositions containing chemically modified wood flour, the highest tensile strength values were recorded for the EP6-WF_IL-1 and EP6-WF_IL-3 compositions (47.99 MPa and 46.83 MPa, respectively). Additionally, the highest relative elongation at break was also characteristic of the EP6-WF_IL-1 composition (2.93%), which, combined with the highest tensile strength result, indicates good mechanical properties of this composite. The lowest relative elongation at break was 2.15% and was recorded for the EP6-WF_IL-2 composition.

The highest value of bending strength and bending modulus of elasticity (Figure 5B and Table 3) was observed for the EP6-WF_IL-2 composition (65.64 MPa and 1855.3 MPa, respectively). Simultaneously, these results were also higher than the value recorded for the reference composition. Additionally, it was found that the material containing unmodified wood flour (EP6-WF) is characterized by the lowest bending strength (47.65 MPa), but a fairly high modulus of elasticity (1744.0 MPa). At the same time, comparing the obtained mechanical results, it was observed that the greatest impact on increasing the strength parameters in the bending range was achieved by modification using ionic liquid IL-2. Modification of wood filler with ionic liquid IL-3 also improved these properties, but to a lesser extent than using IL-2. In turn, the use of IL-1 had the greatest impact on bending modulus. One of the highest values of bending modulus was recorded for the composition EP6-WF_IL-1 (1825.3 MPa). Chemical modification, on the one hand, increases the additive’s compatibility with the polymer matrix. However, as mentioned above, it also affects its morphological appearance and particle size. Analyzing the obtained results of the mechanical tests, it can be noted that the greatest impact on the increase in the value of the flexural elasticity modulus was the introduction of wood particles with sizes 20–115 µm (EP6-WF_IL-1 and EP6-WF_IL-2).

The recorded values of compressive strength (Figure 5C and Table 3) indicate that all tested compositions are characterized by similar strength. The highest value was recorded for the composition without filler, and the lowest for the composition containing unmodified wood flour (67.87 MPa and 60.98 MPa, respectively). At the same time, it was found that among the tested compositions, the highest compression deformation was recorded for the EP6-WF_IL-3 composition (1.92%).

Analyzing the results of the Charpy impact strength without notch and Rockwell hardness (Table 3), it can be seen that both the highest impact strength and hardness were distinguished by the EP6-WF_IL-1 composition (12.02 kJ/m^2^ and 90.70 MPa, respectively). The lowest hardness (69.40 MPa) was recorded for both the EP6-WF and EP6-WF_IL-3 compositions, indicating that these materials are the least resistant to local plastic deformation. Summarizing, the composition with wood flour modified with ionic liquid IL-1 is characterized by the highest ductility and resistance to brittle fracture.

**Figure 6:**
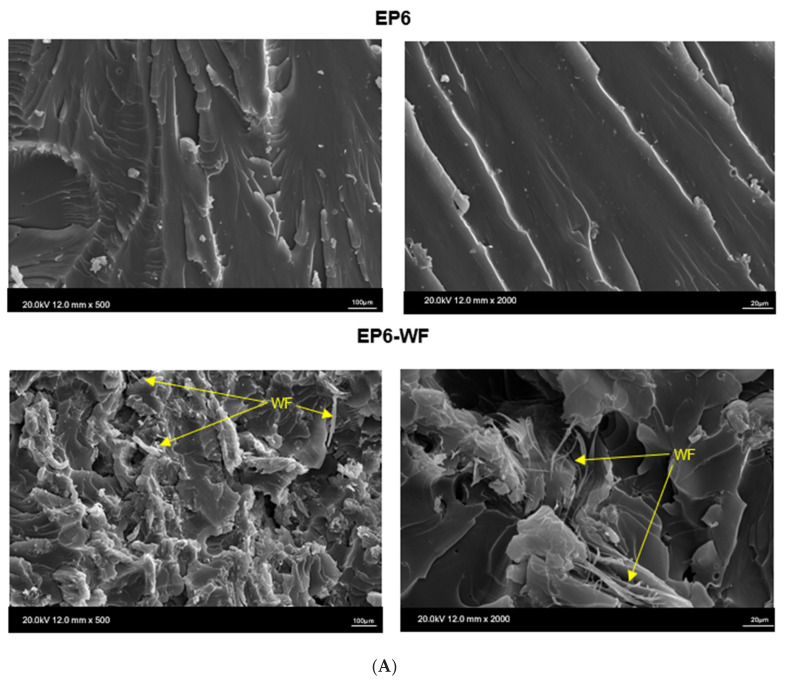

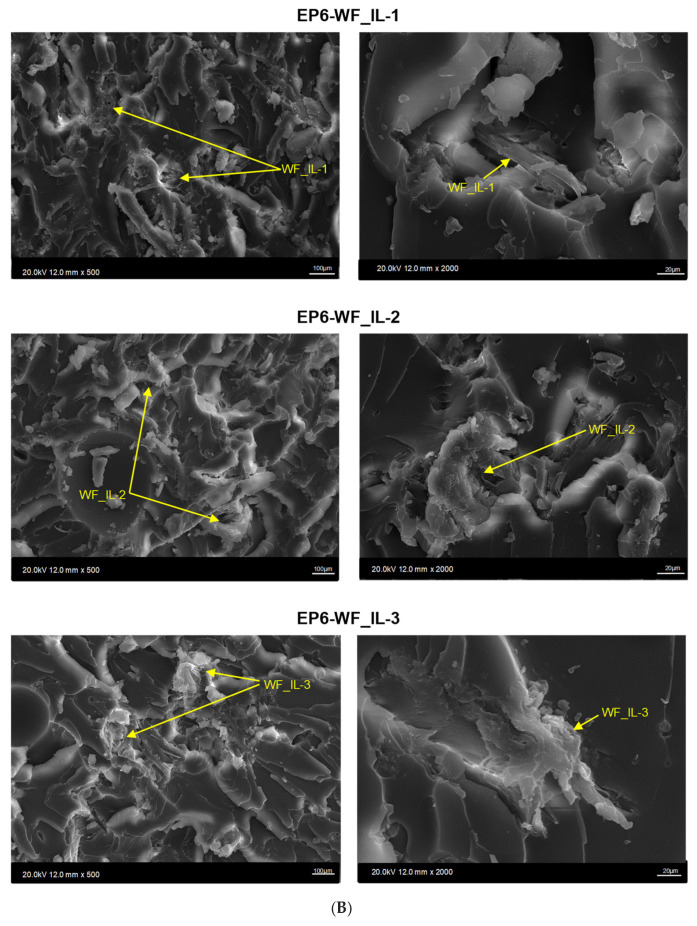
Micrographs of the impact fracture surface of composites based on Epidian 6: (**A**) cured epoxy resin composition without the filler (EP6) and composite with 5 wt.% of unmodified wood flour (EP6-WF), and (**B**) epoxy composites filled with 5 wt.% of wood flour modified by ionic liquids (EP6-WF_IL-1, EP6-WF_IL-2, and EP6-WF_IL-3).

On SEM microimages (Figure 6) of epoxy composites with wood flour modified using ionic liquids, the interface between the composite matrix and filler is noticeable. For all composites, pullouts of wood particles from the polymer matrix are visible. However, comparing the influence of chemical modification of wood flour with ionic liquids on the interaction between fillers and epoxy matrix, it can be observed that wood particles in composites EP6-WF_IL-1, EP6-WF_IL-2, and EP6-WF_IL-3 are more coated with polymer matrix than in the case of pure oak flour [21].

In summary, the introduction of wood flour to the epoxy composition modified with trihexyltetradecylphosphonium bis(2,4,4-trimethylpentyl)phosphinate (EP6-WF_IL-2) resulted in obtaining a composition characterized by the highest values of bending strength and bending modulus. Good strength parameters, including Rockwell hardness, Charpy impact strength, and tensile strength, were also recorded for the EP6-WF_IL-1 composite, containing waste wood flour modified with trihexyltetradecylphosphonium bis(trifluoromethylsulfonyl)amide. It is worth noting that, in comparison to the reference material, for the EP6-WF composite with unmodified fibres, similar or slightly lower values of the tensile and bending modulus, or compressive strength and deformation in compression were recorded. This suggests that, despite the tendency of natural fibres to absorb moisture, they transfer the applied force relatively well, and the polymer matrix protects their surface against mechanical damage, transferring the resulting stresses. Chemical modification of wood filler increases both the additive’s compatibility with the polymer matrix and affects its morphological appearance and particle size, which was confirmed by the improved results of mechanical tests and recorded microscopic images of the obtained compositions.

### 2.3. Enzymatic Degradation of Epoxy Composites Containing Bio-Waste Modified by Ionic Liquids

Since recently much emphasis has been placed not only on the use of raw materials of natural origin, but also on natural methods of material degradation, the next stage of research involved studies on the enzymatic degradation of obtained epoxy materials and the investigation of the influence of the application of wood fillers and their chemical modification on the degradation process. Samples of the obtained epoxy compositions (EP6, EP6-WF, EP6-WF_IL-1, EP6-WF_IL-2, and EP6-WF_IL-3) were subjected to enzymatic degradation for 4 weeks. For this purpose, small samples were prepared with a thickness of approximately 1–2 mm and a mass of 40–90 mg. The incubation of cut-out samples subjected to degradation was carried out using two enzymes, i.e., Porcine Pancreas lipase (10 U/mg, Sigma Aldrich) and Rhizopus Oryzae lipase (100–400 U/mg, Sigma Aldrich) (Table 4).

Lipases catalyze the hydrolysis of esters in aqueous solutions and the synthesis of esters in non-aqueous solutions, as well as the hydrolysis of triacylglycerols to glycerol and free fatty acids [41]. They are used industrially for the separation of chiral compounds and transesterification for biodiesel production [42]. Porcine Pancreas is a lipase obtained from the pancreas of pigs, and its applications include, among others, the analysis of fatty acids or the determination of the indigestible fraction of plant material resistant to the action of digestive enzymes. It also occurs as a component of solubilizing agents for the study of theophylline dissolution profiles [43]. In turn, the Rhizopus Oryzae enzyme is a lipase obtained from the saprotrophic fungus Rhizopus Oryzae, and it is applied in the production of detergents and biodiesel. This lipase is a non-specific enzyme that shows excellent activity over a wide range of pH and temperature values, with the highest thermostability [44]. Enzymes are used for the degradation of several types of plastics [45,46].

**Figure 7:**
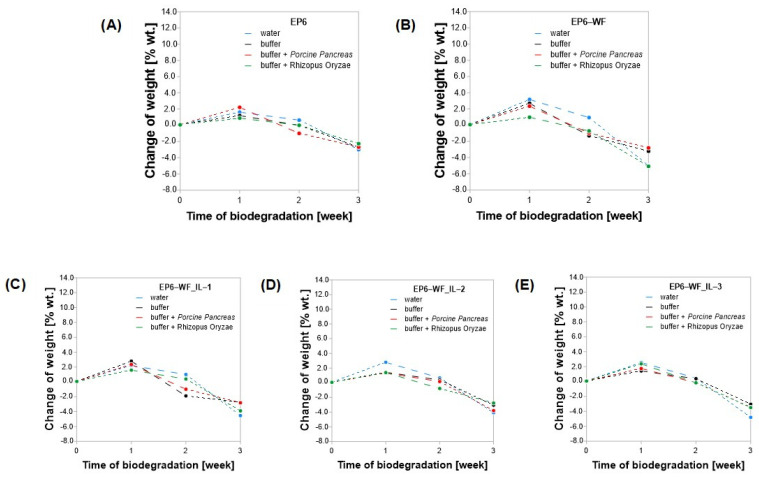
Change in the mass of epoxy samples. (**A**) EP6; (**B**) EP6-WF; (**C**) EP6-WF_IL-1; (**D**) EP6-WF_IL-2; (**E**) EP6-WF_IL-3.

Before subjecting the samples to enzymatic degradation, the FT-IR spectra and microscopic images of the surface, and contact angle measurements were performed. In each subsequent week of incubation, the pH of the buffer solution was measured, and then the samples were washed with methanol and left for a few minutes in a dryer for solvent evaporation. After the analysis of the mass change, FT-IR spectra were recorded. It is worth mentioning that to maintain enzyme activity at the desired level throughout the entire study, each week, the incubation solution, along with the enzyme, was replaced with a new one. After the entire cycle of 28 days of biodegradation, a final set of analyses was performed, including measurement of the pH of the environment, the samples’ weight, their chemical structure, and the morphological and wettability changes in their surface. For all the tested compositions, during the first few days of incubation, relatively rapid water absorption was observed (Figure 7). Water absorption is related to the rate of its penetration into the polymer material. The polymer matrix has sorption capabilities, which means it absorbs water, resulting in the swelling of the polymer occurring as an increase in the mass of the samples. The incubation of the cured pure epoxy sample EP6 in water resulted in an increase in its weight by 1.62% during the first week. Simultaneously, during that time, the greatest mass increase, and thus the greatest differences in the mass of the samples after the first week of incubation, occurred in the composite samples containing a natural filler (EP6-WF—3.16%). The phenomenon of greater water absorption by the composites containing wood flour is related to its stronger absorption by natural fibers, which are largely composed of cellulose. Cellulose molecules have free, polar hydroxyl groups that cause the attraction of water molecules through hydrogen bonds, which in turn results in the accumulation of moisture in the cell wall and is manifested by the swelling of the fibers [47]. Incubation in water of samples containing wood waste modified with ionic liquids also resulted in the highest increase in sample mass during the first week. A corresponding increase by 2.19%—EP6-WF_IL-1, 2.78%—EP6-WF_IL-2, and 2.55%—EP6-WF_IL-3. In order to check the water absorption of the wood waste used to prepare the composites, an additional experiment was carried out. The weighed amount of the unmodified and modified with ionic liquid wood flour was incubated in water for 24 h at room temperature. Then, wood flour was filtered and weighed again. Based on the obtained results, it was found that the unmodified flour was characterized by water absorption at the level of 51.00%, while WF_IL-1—44.97%, WF_IL-2—71.59% and WF_IL-3—50.00%. The ionic liquid IL-1 had the greatest impact on the increase in the hydrophobic properties of wood flour, while the application of IL-2 increased water absorption. The observed phenomenon might be related to the previously mentioned more compact form of WF_IL-2 wood flour. 

For samples incubated in water, regardless of the type of composite, a weekly increase in their weight, caused by swelling of the natural fibers and/or the polymer matrix, was observed. As mentioned earlier, after degradation, the samples were washed with methanol and dried in a dryer for 24 h. Compared to the reference weight, determined before subjecting epoxy samples to degradation, the samples incubated in water showed a mass loss of as much as 1.9 percent in the case of the EP6-WF_IL-3, which may suggest that water also affects the polymer degradation process. Cured epoxy composites are susceptible to moisture absorption and are very brittle, due to their high cross-linking density. Hydrolysis causes cleavage of the carbon bonds of the polymer backbone, which may further disrupt the adhesion between the filler and the polymer matrix [48]. For EP6-WF_IL-3, incubated in buffer solution, a small increase in mass was observed initially, which then remained at a relatively constant level until the 21st day of measurement. Comparing measured samples’ weight to the reference value, the largest overall mass loss in this environment was observed for samples EP6-WF_IL-1 (−1.93%) and EP6-WF (−1.92%). Hydrolytic degradation in the presence of anions and cations has a strong catalytic effect, which may explain the mass loss in the buffer environment [49].

Samples of all compositions incubated in buffer solutions with enzymes Porcine Pancreas or Rhizopus Oryzae are distinguished by an initial increase in mass, related to swelling of the polymer matrix and/or natural fibers of the filler. In the following weeks of measurement, their mass was found to decrease or remain at a constant level. For samples degraded with Porcine Pancreas lipase, on the 21st day of measurement, the largest weight loss (in the range of −0.88% to −0.98%) was observed for the EP6-WF composite samples. Samples incubated with Rhizopus Oryzae on the 21st day of measurement were distinguished by a small mass loss or no changes in this respect. The largest mass loss on this day (−0.55%) was observed for the EP6-WF_IL-2 composite. After 28 days of degradation, the highest total mass loss of samples incubated in buffer and Porcine Pancreas lipase was −2.34% for the EP-WF_IL-2 composition, while in the case of the buffer solution with Rhizopus Oryzae lipase, it was −4.85% for the EP-WF composition.

Summarizing, the greatest sample weight loss, compared to the referenced value determined before performing the enzymatic degradation, was recorded for samples EP-WF (−4.85%), incubated in buffer containing Rhizopus Oryzae lipase. This phenomenon can be explained by the internal pressure on the matrix by natural fibers, caused by the tendency of the fibers to swell and by the limitations of the polymer matrix. The generated internal pressure could have become the cause of internal stresses, which could have resulted in the formation of microcracks in the matrix and ultimately led to mass loss [50]. In the case of a composite containing unmodified wood waste, the hydrophilic properties of the wood filler undoubtedly had the greatest impact on the progression of the erosion process. While in the case of composites with modified waste, increased local erosion processes could be caused by the accumulation of larger particles of the lignocellulosic additive. It can be concluded that the chemical modifications of the surface of waste wood flour using ionic liquids of different structures have an impact on the enzymatic degradation of the obtained composite material. For both applied enzymes, the greatest weight loss of epoxy composite samples containing modified fibers was observed in the case of the composite containing wood flour modified with ionic liquid IL-2, suggesting that trihexyltetradecylphosphonium bis(2,4,4-trimethylpentyl)phosphinate used for modification could increase the susceptibility of the polymer material to degradation. The smallest weight loss, regardless of the type of applied enzyme, occurred in the case of the reference composition without wood filler, which indicates greater strength and lower susceptibility to degradation of the unfilled epoxy composition.

**Figure 8:**
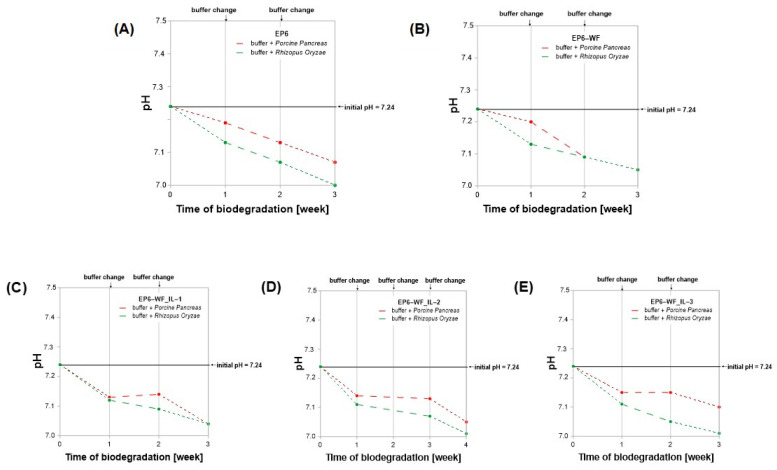
Change in pH of the incubation solutions of the performed enzymatic degradation in the presence of lipases Porcine Pancreas and Rhisopus Oryzae. (**A**) EP6; (**B**) EP6-WF; (**C**) EP-WF_IL-1; (**D**) EP-WF_IL-2; (**E**) EP-WF_IL-3.

The pH of the buffer solutions in which the epoxy samples were subjected to degradation changed over time (Figure 8), along with the progression of degradation of epoxy materials. For the first 7 days, for all buffer solutions, the pH decreases almost linearly, and then in the following days (up to the 21st day) it remains practically at the same level, until it decreases again in the last week of degradation. This may suggest the ongoing hydrolysis of ester bonds, leading to the release of acidic degradation products [49]. It is worth noting that during the degradation process, the enzyme first diffuses from the solution to the surface of the polymer material. It is then adsorbed on the surface, catalyzing the degradation reaction, and the degradation products are finally released into the solution, affecting the change in the pH of the environment [51].

After the first week of incubation of the degraded composite samples, the largest pH drop was observed in the case of the environment containing the Rhizopus Oryzae enzyme, with the largest change noted for the EP6-WF_IL-2 and EP6-WF_IL-3 composites (from pH 7.24 to 7.11). In comparison of the results obtained for phosphorus buffers with RO with the values registered for the solutions containing Porcine Pancreas, the largest drop was observed for the EP6-WF_IL-1 composite, amounting to 7.14. After the 21st day of incubation, the largest pH change (by 0.1 units) was noted for the EP6-WF composition samples incubated in the buffer and the Porcine Pancreas enzyme. In the case of degradation using Rhizopus Oryzae, the largest drop (by 0.06 units) was observed for the EP6-WF_IL-3 composition samples.

The greatest decrease in pH, compared to the initial and final values, was noted for the EP6 composition samples incubated in buffer and Rhizopus Oryzae, with a pH change from 7.24 to 7.00. A clear decrease in pH of the samples incubated in this environment was also observed for the EP6-WF_IL-2 composition (from pH 7.24 to 7.01). The smallest change was noted for EP6-WF_IL-3 composition samples incubated in buffer and Porcine Pancreas enzyme, from pH 7.24 to 7.11.

In general, most buffer solutions of composite samples incubated with Rhizopus Oryzae lipase showed a greater decrease in the pH of the reaction medium than those with the Porcine Pancreas enzyme. It may indicate greater degradation capacity of this enzyme. In addition, changes in pH values were different depending on the composite material subjected to degradation. Hence, it can be assumed that the type of chemical modification to which the natural filler was subjected before application in epoxy composites impacted the biodegradation process. It was observed that this process occurred the fastest for a composite with wood flour modified with trihexyltetradecylphosphonium bis(2,4,4-trimethylpentyl)phosphinate (IL-2). Additionally, regardless of the enzyme used, for samples EP6-WF_IL-2, the largest decrease in sample weight was noted. It may indicate poor binding properties between the filler and the matrix EP6-WF_IL-2, and thus greater susceptibility to enzymatic degradation. It is worth noting that the composition EP6-WF_IL-2 of all the compositions containing wood flour modified with ionic liquids was characterized by slightly lower values of tensile and compressive strength.

**Figure 9:**
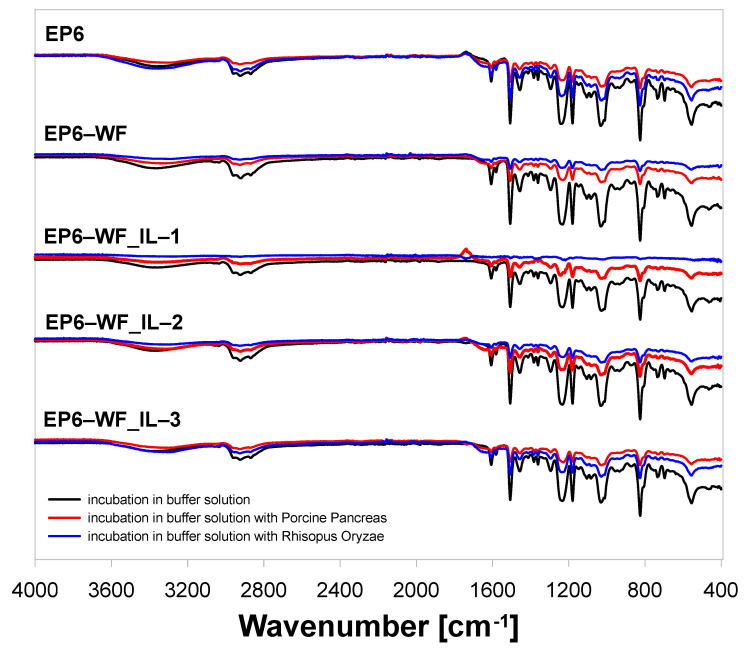
FT-IR spectra of the epoxy samples subjected to enzymatic degradation.

Comparing the FT-IR spectra recorded before degradation and those after four weeks of incubation in a buffer containing Porcine Pancreas or Rhizopus Oryzae enzymes (Figure 9), one can observe a relatively lower intensity of the signal characteristic of the ester group vibrations at v = 1605 cm^−1^. The lower intensity of this signal may suggest partial hydrolysis of the ester group [52]. Additionally, a lower intensity of the band in the range of 3200–3600 cm^−1^, characteristic of the stretching vibrations of the -OH group, was also found. It is also worth noting that, as a result of the degradation, no significant changes were observed in terms of the shift in the vibration areas. In turn, the greatest changes in the vibration intensity were observed in the case of EP6-WF_IL-1, which may suggest greater susceptibility to degradation of composite materials with wood fillers modified with ionic liquid IL-1. On the other hand, the smallest changes were found in the spectra recorded for the unfilled epoxy composition. In addition, it was noted that for samples degraded in a buffer solution containing Porcine Pancreas, a greater decrease in signal intensity was recorded than in the case of those compositions treated with the enzyme Rhizopus Oryzae.

**Figure 10:**
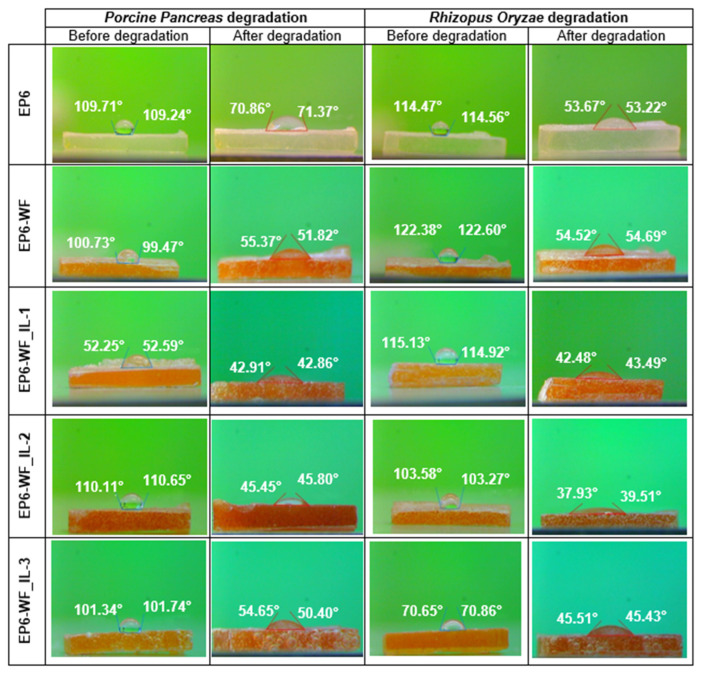
The average value of the contact angle before and after the biodegradation process.

The contact angle values recorded before and after sample incubation (Figure 10) indicate a significant change in the wettability of the surfaces of the tested samples. For samples degraded in a buffer environment containing the Porcine Pancreas enzyme, the greatest decrease in the wetting angle value was observed for the EP6-WF_IL-2 composite (62.1% compared to the initial values) and the EP6-WF_IL-3 composite (51.4%). The smallest decrease in the wetting angle value was observed for the reference composition, approximately by 20% on average. In turn, for samples degraded in a buffer environment containing the Rhizopus Oryzae enzyme, the greatest total decrease, approximately by 66.9% in the wetting angle, was observed for the EP6-WF_IL-1 composite. The recorded value was also the largest change noted in the entire study. A significant decrease was also observed for the EP6-WF_IL-1 composite (54.7%) and EP6-WF (53.2%), and the smallest for the EP6-WF_IL-3 composite (an average of 43.5%). For the EP6-WF_IL-3 composite, the smallest change in pH of the degradation environment was also noted. It may suggest that the composite modified with IL-3 ionic liquid as a result of the applied treatment shows better interfacial adhesion between the polymer matrix and the fiber, and is, therefore, less exposed to enzymatic hydrolysis.

The observed changes in the wetting angle indicate that the enzymatic degradation resulted in a decrease in the hydrophobicity of the polymer material. At the same time, it was found that the highest wetting angle was recorded on the rough composite surfaces, while the lowest was obtained for smooth samples. Based on the analysis of the results in surface wettability, it can be assumed that the Rhizopus Oryzae enzyme demonstrates greater degradation activity of epoxy materials filled with waste wood flour.

**Figure 11:**
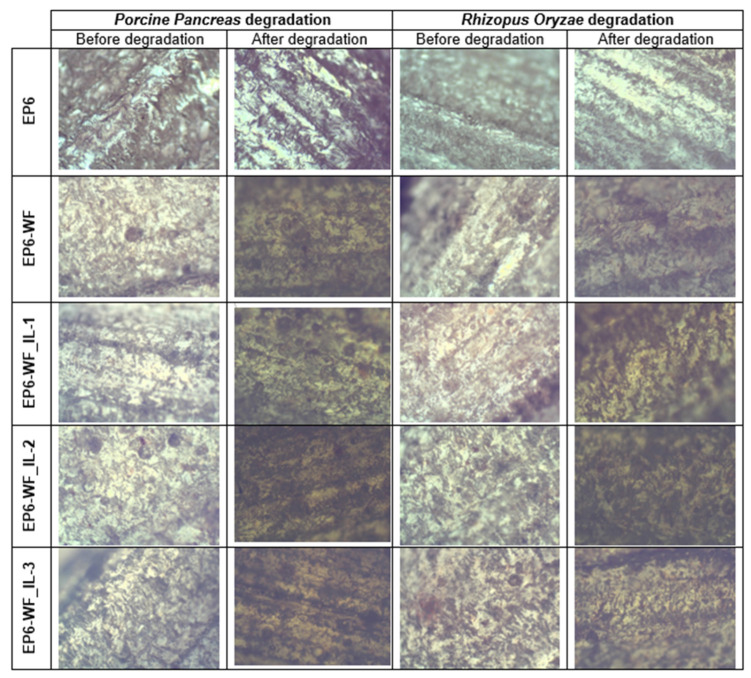
Microscopic analysis of selected samples (a 4× magnification images of composite samples subjected to enzymatic degradation).

The images recorded using the optical microscope (Figure 11) indicate the leaching of the wood filler from the polymer matrix during enzymatic degradation. Moreover, there are visible minor damages and numerous cracks on the surface of the samples. The surface of the samples before enzymatic degradation seems relatively stiff and smooth, and does not contain any visible cracks. As a result of the action of enzymes, the surface became rougher with numerous small surface defects. In addition, the progress of enzymatic degradation is visible in a change in the color of the tested samples, which became much darker. In general, the degradation process significantly weakened the interfacial adhesion between the matrix and the filler, and thus adversely affected the properties of the composites. Comparing the images taken for the reference composition and the composites reinforced with modified natural fibers, it can also be assumed that the introduction of a natural filler will accelerate the degradation of the composite when the material is exposed to the action of enzymes [53].

**Figure 12:**
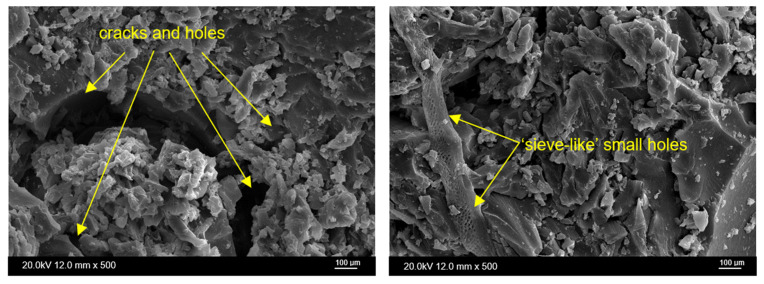
Microimages of post-enzymatic degradation of the EP6-WF sample.

The erosion of the surface due to enzymatic degradation is visible on the microimages presented in Figure 12. On the surface of EP6-WF, several cracks and jagged small fragments protrude from the composition. It is possible to observe larger uniform fragments, as well as sieve-like small holes.

In summary, the results of the enzymatic biodegradation showed that the greatest mass change occurred in the case of compositions containing unmodified wood flour—EP6-WF incubated in buffer and Rhizopus Oryzae. In addition, greater pH changes were found for composites incubated with Rhizopus Oryzae lipase, compared to those with Porcine Pancreas. It indicates greater degradation capabilities and thus greater effectiveness of this enzyme in the degradation process of the considered composite materials. The largest changes in the contact angle occurred for the EP6-WF_IL-1 composite, incubated with the Rhizopus Oryzae enzyme, and the smallest for EP6-WF_IL-3, in the same degradation environment. In addition, the smallest change in pH of the buffer solution containing Porcine Pancreas lipase was recorded for EP6-WF_IL-3, suggesting that this material is the least susceptible to degradation, regardless of the enzyme used. It is also worth highlighting that in the case of a composite containing unmodified wood waste, the hydrophilic properties of the wood filler had a significant impact on the progression of the erosion process of the material. It increased the accessibility of the surface of the material to the influence of the enzyme contained in the incubation solution. While in the case of composites with modified waste, increased local erosion processes could be caused by the accumulation of larger particles of the lignocellulosic additive.

## 3. Materials and Methods

Materials. Epidian 6 (EP6, low-molecular-weight bisphenol A epoxy resin, EV = 0.536 mol/100 g, CIECH Sarzyna S.A., Nowa Sarzyna, Poland), IDA hardener (amine hardener based on isophorone diamine for liquid epoxy resins, CIECH Sarzyna S.A.), the oak wood waste flour (WF, FHU Parkiety Smolik, Lanckorona, Poland), Tetradecyltrihexylphosphonium bis(trifluoromethylsulfonyl)amide (IL-1, Sigma Aldrich, Poznan, Poland), Tetradecyltrihexylphosphonium bis(2,4,4-trimethylpentyl)phosphinate (IL-2, Sigma Aldrich), and 1-Ethyl-3-methylimidazolium bis(trifluoromethylsulfonyl)imide (IL-3, Sigma Aldrich); Enzymes: Porcine Pancreas lipase (10 U/mg, Sigma Aldrich) and Rhizopus oryzae lipase (100–400 U/mg, Sigma Aldrich), Na_2_HPO_4_ (Sigma Aldrich), KH_2_PO_4_ (Sigma Aldrich), NH_4_Cl, (Sigma Aldrich), MgSO_4_·7H_2_O (Sigma Aldrich), NaCl (Sigma Aldrich), H_3_BO_3_ (Sigma Aldrich), CuSO_4_·5H_2_O (Sigma Aldrich), FeCl_3_·6H_2_O (Sigma Aldrich), ZnCl_2_ (Sigma Aldrich), MnSO_4_·5H_2_O (Sigma Aldrich), (NH_4_)_5_Mo_7_O_24_·7H_2_O (Sigma Aldrich).

### 3.1. Preparation of Waste Wood Filler

Initially, waste oak flour, obtained as raw waste from processing boards intended for wooden floors, was separated into individual fractions using a laboratory shaker for sieve analysis. The wood waste consisted of particles of non-uniform sizes: >0.2 mm; 0.2–0.16 mm; 0.16–0.1 mm; 0.1–0.076 mm; 0.076–0.056 mm; 0.056–0.04 mm, and <0.04 mm. A fraction of wood flour with a particle size of <0.04 mm was subjected to further modification and epoxy composites preparation. The chemical modification of the wood waste and its application as a filler for epoxy composites were preceded by a 48 h pre-drying stage at a temperature of 80 °C. Next, 10 g of wood waste with a particle size of <0.04 mm was placed in a beaker and thoroughly mixed with 10 wt.% of selected ionic liquid (trihexyltetradecylphosphonium bis(trifluoromethyl-sulfonyl)amide, trihexyltetradecylphosphonium bis(2,4,4-trimethylpentyl)-phosphinate, or methyltrioctylammonium bis(trifluoromethylsulfonyl)imide), followed by the addition of 50 cm^3^ of ethanol. Such a mixture was incubated for 3 h at the lab temperature. Then the modified wood waste flour was filtered using a Büchner funnel under reduced pressure and left to dry in a dryer at 60 °C for 24 h.

### 3.2. Preparation of Epoxy-Waste Wood Composites

Low-molecular-weight epoxy resin, Epidian 6, was mixed (for 30 min, maintaining the mixer speed at 700–800 rpm) with unmodified or chemically modified wood waste in an amount corresponding to 5 wt.%, and deaerator BYK A530 (1 wt.% of the total composition). Then, the amine hardener IDA was added in an amount calculated based on the equation, including the content of epoxy functional groups in the epoxy resin ($m_{hardener}=EV\cdot 95$, where $m_{hardener}$ is the amount [g] of IDA amine hardener to crosslink 100 g of epoxy resin, and EV is the epoxy value [mol/100 g epoxy resin], the amount of epoxy functional groups in Epidian 6 epoxy resin). All compositions were intensively mixed with a baguette until a homogeneous consistency was obtained. Then each mixture was deaerated for 3 min at a pressure of 0.6 MPa, poured into Teflon molds, and left to harden at room temperature for 24 h. After 24 h, the finished samples (in the form of paddles, beams, and rollers) used for further mechanical tests were removed from the molds and conditioned at room temperature for 7 days, followed by additional cross-linking at 80 °C for 24 h.

### 3.3. Biodegradation of Epoxy-Wood Composites

Samples of obtained epoxy-wood composites were subjected to enzymatic degradation for 28 days, in 30 °C, using Rhizopus Oryzae lipase or Porcine Pancreas in phosphate buffer PB with pH = 6.8, composed of Na_2_HPO_4_ (7.0 g/mol), KH_2_PO_4_ (3.0 g/mol), NH_4_Cl, (1.0 g/mol), MgSO_4_·7H_2_O (0.25 g/mol), NaCl (0.5 g/mol), H_3_BO_3_ (0.5 μg/mol), CuSO_4_·5H_2_O (40.0 μg/mol), FeCl_3_·6H_2_O (0.2 μg/mol), ZnCl_2_ (0.4 μg/mol), MnSO_4_·5H_2_O (0.4 μg/mol), (NH_4_)_5_Mo_7_O_24_·7H_2_O (0.2 μg/mol) and distilled water. Each week, 0.5 mg of enzyme per polymer sample was applied within the biodegradation process. Samples of epoxy composites were cut into cuboids with an average size of 0.5 × 1.0 × 0.2 cm and a weight ranging from 0.02 to 0.09 g. The bio-composites were then cleaned through repeated washing with methanol, dried, and initially analyzed before the incubation in buffer solutions in the presence of lipase enzymes. Dried epoxy samples were placed in screw-cap glass vials with a capacity of 4 cm^3^. Three samples of each composition were cut out, incubated in separate vials, and the analyzed results were averaged. The tested epoxy samples, subjected to biodegradation, were comprehensively analyzed by monitoring the changes in the pH of the environment, weight of the polymer sample, its surface morphology, contact angle, and chemical structure. The mentioned tests were performed before, on every 7th day of each cycle, and after the biodegradation process.

### 3.4. Composite Materials’ Characteristics

#### 3.4.1. Spectroscopic Analysis (ATR/FT-IR)

The analysis of the chemical structure of unmodified and modified wood flour, as well as epoxy composite samples before and after the enzymatic degradation, was performed using the Fourier Transform Infrared Spectroscopic method (FT-IR), performed with the SPECTRUM 65 FT-IR apparatus equipped with an ATR attachment (ZeSe crystal with a spectral range of 4000–400 cm^−1^). The measurements were carried out at room temperature. A small amount of loose wood waste or a solid composite sample was placed onto the ZnSe crystal attenuated total reflectance unit (ATR), and it was pressed down with a screw equipped with an attachment. The obtained spectra were presented as a function of transmittance T [%] from the wavenumber v [cm^−1^] in the range of 4000–600 cm^−1^.

#### 3.4.2. The Mechanical Properties of Epoxy-Wood Composites

The mechanical properties of the prepared epoxy-wood composites were tested according to previously described procedures [21], including plastics-determination of tensile properties—PN-EN ISO 527-1:2012 [54], plastics-determination of flexural properties—PN-EN ISO 178:2011 [55], plastics-determination of compressive properties—PN-EN ISO 604:2006 [56], and plastics-determination of Charpy impact properties—PN-EN ISO 1792:2001 [57]. Tests were performed using samples of epoxy-wood composites in the shape of paddles, beams, and rollers. The prepared compositions were tested on the Zwick 1445 apparatus (tensile strength, elongation at break, modulus elasticity, flexural strength, elasticity, flexural modulus, compressive strength, and compression set) and ZORN PSW 4J Digital apparatus (the impact strength without notches by the Charpy method).

#### 3.4.3. Morphological Analysis

Morphological analysis was performed using a metallographic microscope with a Delta Optical MET-200 camera (Delta Optical, Nowe Osiny, Poland) (composite samples subjected to enzymatic degradation) and a JEOL JSM 6010LA scanning electron microscope (JEOL, Tokyo, Japan, micro images of the impact-fractured surfaces of the epoxy-wood composites and pre-dried wood flour particles). The images recorded using an optical microscope were taken at approximately 4× magnification. SEM microphotographs were recorded at a 5 kV acceleration using samples coated with a thin film of gold.

#### 3.4.4. Contact Angle Measurements

Wettability tests were carried out using a set equipped with a microsyringe filled with distilled water and a camera for recording images of drops of liquid applied to the test sample. The captured images were analyzed using the MicroCapture-DNT 2.0 program.

## 4. Conclusions

Chemical modification of wood flour waste from sanding oak floors was carried out. For the functionalization of lignocellulosic biowaste, three various ionic liquids (trihexyltetradecylphosphonium bis(trifluoromethylsulfonyl)amide, trihexyltetradecyl-phosphonium bis(2,4,4-trimethylpentyl)phosphinate, and methyltrioctylammnium bis(trifluoromethylsulfonyl)imide) were used. Based on the analysis of the recorded FT-IR spectra of unmodified wood flour and lignocellulosic waste after modification with ionic liquids, it was found that in comparison with the spectrum of unmodified wood flour, in the spectrum of flour modified with ionic liquids, a decrease in the intensity of the signal characteristic for the stretching vibrations of the acetyl group of lignin (v = 1227 cm^−1^) was observed, most likely indicating partial removal of lignin from wood flour. Moreover, the spectra of wood flour modified with ionic liquids showed characteristic signals for the respective ionic liquids, i.e., vibrations of the amide group at v = 2915 cm^−1^ and v = 2845 cm^−1^ for WF_IL-1 flour and vibrations of the imide group at v = 2935 cm^−1^ and 2850 cm^−1^ for WF_IL-3 flour.

Five different epoxy compositions based on low-molecular-weight epoxy resin Epidian 6 were obtained, including a reference composition without filler and compositions containing 5% by weight of unmodified wood flour and chemically modified wood flour. Based on the tests of selected mechanical properties of the obtained epoxy materials, it was found that among the samples containing wood flour, the EP6-WF_IL-2 composition was characterized by the highest values of bending strength and flexural modulus (65.64 MPa and 1855.3 MPa, respectively). At the same time, the measured value of bending strength was 1.91 MPa higher than that recorded for the reference composition (65.64 MPa—EP6-WF_IL-2 compared to 63.73 MPa—EP6).

As a result of the enzymatic degradation of the obtained epoxy compositions using the Porcine Pancreas lipase and Rhizopus Oryzae lipase enzymes, it was found that the largest overall mass loss was noted for the EP6-WF composite samples (−4.85%) incubated in a buffer containing the Rhizopus Oryzae enzyme. As the degradation progressed, a change in the pH of the incubation solutions was also observed. In the first 7 days, a rapid drop in the pH value was noted, which then (until the 21st day) remained at a generally constant level, which may indicate the ongoing processes of hydrolysis of ester bonds with the release of acidic decomposition products that affect the change in the pH value of the reaction environment. The post-degradation microscopic analysis of epoxy composite samples showed increased surface roughness and the formation of additional minor damage and cracks. In addition, the contact angle measured for all samples subjected to enzymatic degradation was less than 90°, indicating an increase in the hydrophilic properties of the surface of the degraded composites. The largest change (by 66.9%) was observed for the EP6-WF_IL-1 composite incubated in a buffer containing the Rhizopus Oryzae enzyme. For the same conditions of enzymatic degradation, the smallest value (by 43.5%) was recorded for the EP6-WF_IL-3. Considering the mentioned results in combination with the smallest change in pH of the buffer solution containing EP6-WF_IL-3 and Porcine Pancreas, it can be stated that samples containing wood flour modified using methyltrioctylammonium bis(trifluoromethylsulfonyl)imide show the lowest susceptibility to enzymatic degradation, regardless of the degradation environment.

## Figures and Tables

**Table 1:** FT-IR analysis of unmodified and ionic liquid-modified wood flour.

Signal	Frequency [cm^−1^]				Associated Band
	Unmodified Wood Flour WF	Wood Flour Chemically Modified with Ionic Liquids			
		WF_IL-1	WF_IL-2	WF_IL-3	
1	3100–3570	3095–3575			valence stretching -OH and/or -N-H
2	2899	2915, 2845	2865	2935, 2850	valence stretching -C-H
3	1724	1722	1722	1718	valence stretching -C=O
4	1592, 1470	1590, 1467			stretching C=C of the ring, skeletal vibrations in the plane of the ring
5	1417	1418	1420	1420	symmetrical bending -CH_2_
6	1363, 1314	1328, 1320	1363, 1312	1354, 1322	bending -C-H and -C-O groups of the aromatic ring/stretching -C-N of the imide group
7	1227	-	1227	-	stretching -C-O of the acetyl group
8	-	1199	-	-	stretching -C-O-C of the amide group
9	-	-	-	1185	bending H-C-C and H-C-N of the imide ring
10	1080	1085, 1045	1085	1080, 1040	stretching -C-O-C
11	1022	1025			stretching -C-O ether group
12	888	891			β-glycosidic bonds between monosaccharides
13	-	620–500	-	620–500	deformation of the amide/imide ring
14	560	556	552	556	bending -C-OH

**Table 2:** Composition of epoxy-wood composites.

Epoxy Composition	Epoxy Resin	Hardener	Deaerator [1 wt.%]	Filler [5 wt.%]
REF	Epidian 6	IDA hardener	BYK A530	-
EP6-WF				Unmodified wood flour
EP6-WF_IL-1				Wood flour modified with IL-1
EP6-WF_IL-2				Wood flour modified with IL-2
EP6-WF_IL-3				Wood flour modified with IL-3

**Table 3:** Mechanical properties of epoxy compositions based on Epidian 6 filled with waste wood flour.

Mechanical Properties	Epoxy Compositions Based on Low Molecular Epoxy Resin Epidian 6				
	Epoxy Composition Without the Filler	Epoxy Composites with Unmodified Wood Flour	Epoxy Composites with Wood Flour Modified with Ionic Liquids		
	REF	EP6_WF	EP6-WF_IL-1	EP6-WF_IL-2	EP6-WF_IL-3
Modulus of elasticity (MPa)	2441.18 ± 227.55	839.45 ± 121.90	814.97 ± 60.22	698.90± 114.00	951.90 ± 47.94
Elasticity flexural modulus (MPa)	1628.0 ± 59.1	1744.0 ± 82.0	1825.3 ± 92.7	1855.3 ± 140.5	1551.0 ± 142.8
Rockwell Hardness (MPa)	72.0 ± 0.02	69.40 ± 0.03	90.70 ± 0.01	77.80 ± 0.01	69.40 ± 0.01
Impact toughness (kJ/m^2^)	9.15 ± 0.94	10.26 ± 3.48	12.02 ± 2.12	9.21 ± 0.92	7.13 ± 1.71

**Table 4:** Conditions of enzymatic degradation of epoxy compositions.

Parameter	Conditions of Enzymatic Degradation	
Temperature	30 °C	
pH	7.24	
Time of degradation	30 days	
Type of enzyme	Porcine Pancreas	Rhizopus Oryzae
Concentration of the enzyme	0.5 mg/week	0.5 mg/week
Buffer solution	NH_4_Cl—1.0 g/L MgSO_4_·7H_2_O—0.25 g/L Na_2_HPO_4_—7.0 g/L NaCl—0.5 g/L H_3_BO_3_—0.5 µg/L CuSO_4_·5H_2_O—40.0 µg/L FeCl_3_·6H_2_O—0.2 µg/L ZnCl_2_—0.4 µg/L MnSO_4_·5H_2_O—0.4 µg/L (NH_4_)_5_Mo_7_O_24_·7H_2_O—0.2 µg/L	

## Data Availability

Data available on request.

## Abbreviations

The following abbreviations are used in this manuscript:

IL-1	trihexyltetradecylphosphonium bis(trifluoromethylsulfonyl)amide
IL-2	trihexyltetradecylphosphonium bis(2,4,4-trimethyl-pentyl)phosphinate
IL-3	methyltrioctylammonium bis(trifluoromethylsulfonyl)imide
PP	Porcine Pancreas lipase
RO	Rhizopus Oryzae enzyme
REF	Cured epoxy composition based on low-molecular-weight epoxy resin Epidian 6
WF	Cured epoxy composite based on low-molecular-weight epoxy resin Epidian 6 filled with 5 wt.% of unmodified wood flour
EP-WF_IL-1	Cured epoxy composite based on low-molecular-weight epoxy resin Epidian 6 filled with 5 wt.% of wood flour modified with trihexyltetra-decylphosphonium bis(trifluoromethyl-sulfonyl)amide
EP-WF_IL-2	Cured epoxy composite based on low-molecular-weight epoxy resin Epidian 6 filled with 5 wt.% of wood flour modified with trihexyltetradecylphosphonium bis(2,4,4tri-methyl)pentylphosphinate
EP-WF_IL-3	Cured epoxy composite based on low-molecular-weight epoxy resin Epidian 6 filled with 5 wt.% of wood flour modified with methyltrioctylammonium bis(trifluoromethyl-sulfonyl)imide
